# Editorial: Natural compounds in veterinary therapeutics

**DOI:** 10.3389/fvets.2026.1775123

**Published:** 2026-01-19

**Authors:** Baocheng Hao, Dongan Cui, Shuaiyu Wang

**Affiliations:** 1 Key Laboratory of New Animal Drug Project of Gansu Province, Lanzhou, China; 2 Key Laboratory of Veterinary Pharmaceutical Development, Ministry of Agriculture and Rural Affairs, Lanzhou, China; 3 Lanzhou Institute of Husbandry and Pharmaceutical Sciences of Chinese Academy of Agriculture Sciences, Lanzhou, China; 4 College of Veterinary Medicine, Lanzhou University, Lanzhou, China; 5 College of Veterinary Medicine, China Agricultural University, Beijing, China

**Keywords:** immunomodulation, intestinal health, natural compounds, pharmacologic mechanism, safety evaluations, veterinary therapeutics

Natural compounds represent a vital source for the discovery of novel drugs (1). Amidst the shift of animal husbandry from traditional practices to large-scale intensive farming, the emergence of increased antibiotic resistance, novel diseases, and mixed infections has intensified the challenges faced by drug prophylaxis and treatment (2). In this context, the screening and discovery of new natural products with therapeutic potential, and the elucidation of their mechanisms of action, have become crucial strategies to address these challenges. A systematic review of the 14 research articles in this Research Topic clearly demonstrates the innovations of natural products in various dimensions, including animal bacterial diseases, viral diseases, intestinal health, immunomodulation, and safety evaluations, offering new solutions for disease prevention and treatment in modern animal husbandry.

In the field of animal disease prevention and treatment, natural products have exhibited remarkable antibacterial and anti-inflammatory activities. Extracts from *Eucalyptus globulus* Leaf, asiatic acid, and ursolic acid have shown significant antibacterial and anti-biofilm activity against mastitis isolates in dairy cows, particularly inhibitory effects on drug-resistant strains, providing alternative solutions to the problem of antibiotic resistance (Mezzasalma et al.). Linalool has been found to disrupt *E. coli* biofilms by dual inhibition of motility and adhesion, pointing the way for the development of anti-biofilm drugs with multi-target mechanisms. Extracts from *Pulsatilla chinensis* have effectively alleviated *Staphylococcus aureus*-induced mastitis in mice by regulating inflammatory responses and the intestinal microbiota, offering a new approach for the prevention and treatment of dairy cow mastitis (Xiang et al.).

Natural products have also shown impressive performance in antiviral and immune regulation. *Scutellaria baicalensis Georgi* extract against has demonstrated antiviral activity against Getah virus both in *vitro* and in *vivo* (Liu B. et al.); Normal butanol fraction of *Polygonum hydropiper* L. flavonoids has reduced inflammation caused by PCV2 infection in cell cultures and mouse models, providing new candidate drugs for the prevention and treatment of viral diseases (Wei et al.). *Glycyrrhiza* Polysaccharides have shown therapeutic potential against pseudorabies virus infection through immunomodulation, antioxidant activity, and restoration of the intestinal microbiota, reflecting the multifaceted and multi-pathway characteristics of natural products (Song et al.). The extract of *Gynostemma Pentaphyllum* has enhanced mucosal immunity against porcine epidemic diarrhea virus, offering a new strategy for the immunoprophylaxis of intestinal virus infections.

Intestinal health is a fundamental aspect of animal health, and research into natural products in this domain is deepening. The Zengye granules have shown a good laxative effect by regulating the SCF/c-Kit pathway and the intestinal microbiota in constipated mice, providing a new option for the treatment of functional constipation (Lv et al.). The study on the gastrointestinal microbiota and serum metabolomics of subclinical mastitis in dairy cows treated with *Astragali Radix* water decoction has revealed the scientific basis for the therapeutic effects of traditional Chinese medicine through the regulation of the intestinal microecology and metabolic networks (Yan et al.).

The safety evaluation of natural products is a critical step before their application. The extract of *Stevia rebaudiana* extract has been confirmed as safe after acute, sub-chronic, genetic, and teratogenicity evaluations, providing a scientific basis for the development of feed additives (Li et al.).

Important progress has also been made in the field of antitumor research. Pseudolaric acid B induces G2/M phase arrest in canine mammary tumor cells by targeting CDK1, offering not only a new strategy for the treatment of canine mammary tumors but also a reference for human breast cancer research (Chen et al.). This study highlights the value of natural products in the era of precision medicine. Moreover, natural products have a unique advantage in the prevention and control of mycotoxin contamination. Research progress on the prevention and treatment of animal Zearalenone poisoning by natural products has provided green solutions to the issue of feed mycotoxin contamination, which is of significant importance for ensuring animal health and food safety ((Liu N. et al).

As research deepens and technology advances, natural products are certain to play an even more critical role in safeguarding animal health, promoting sustainable development in animal husbandry, and ensuring food safety (3). The guest editorial team extends its gratitude to all the reviewers for their diligent efforts in upholding the pursuit of science, advancing its frontiers, and spreading scientific knowledge. The team will continue to adhere to a scientific, rigorous, and pragmatic academic attitude, actively contributing to global scientific and technological progress.
